# Resting-state functional connectivity and structural differences between smokers and healthy non-smokers

**DOI:** 10.1038/s41598-024-57510-3

**Published:** 2024-03-22

**Authors:** Carmen Weidler, Chiara Gramegna, Dario Müller, Maike Schrickel, Ute Habel

**Affiliations:** 1 Department of Psychiatry, Psychotherapy and Psychosomatics, Faculty of Medicine, RWTH Aachen, Pauwelsstraße 30, 52074 Aachen, Germany; 2 PhD Program in Neuroscience, School of Medicine and Surgery, University of Milano-Bicocca, Monza, Italy; 3 Institute of Neuroscience and Medicine, JARA-Institute Brain Structure Function Relationship (INM 10), Research Center Jülich, Jülich, Germany; 4 Department of Psychology, University of Milano-Bicocca, Piazza dell’Ateneo Nuovo 1, 20126 Milan, Italy

**Keywords:** Neuroscience, Cognitive neuroscience

## Abstract

Previous studies have shown an association between cigarette use and altered resting-state functional connectivity (rsFC) in many large-scale networks, sometimes complemented by measures of cortical atrophy. In this study, we aimed to further explore the neural differences between smokers and healthy non-smokers through the integration of functional and structural analyses. Imaging data of fifty-two smokers and forty-five non-smokers were analyzed through an independent component analysis for group differences in rsFC. Smokers showed lower rsFC within the dorsal attention network (DAN) in the left superior and middle frontal gyrus and left superior division of the lateral occipital cortex compared to non-smokers; moreover, cigarette use was found to be associated with reduced grey matter volume in the left superior and middle frontal gyrus and right orbitofrontal cortex, partly overlapping with functional findings. Within smokers, daily cigarette consumption was positively associated with increased rsFC within the cerebellar network and the default mode network and decreased rsFC within the visual network and the salience network, while carbon monoxide level showed a positive association with increased rsFC within the sensorimotor network. Our results suggest that smoking negatively impacts rsFC within the DAN and that changes within this network might serve as a circuit-based biomarker for structural deficits.

## Introduction

Cigarette smoking is a major public health issue and the single most preventable cause of death and disease in the world^1^. According to the World Health Organization (WHO), tobacco consumption is related to over 8 million deaths a year. As reported by the study German Health Update (GEDA 2019/2020-EHIS), 28.9% of adults in Germany smoke at least occasionally (24% among women and 33.9% among men)^2^. A recent increase in smoking has also been documented among teenagers in Germany; as of 2022, 15.9% of adolescents between 14 and 17 years old reportedly smoke^3^. Smoking is associated with an increased incidence of a large number of diseases, including cancer, chronic obstructive pulmonary disease, coronary heart disease, stroke, peripheral vascular disease, and peptic ulcer disease^4^. Previous studies have also suggested an association between smoking and volumetric measures of brain atrophy^5,6^; specifically, it has been reported that smokers tend to have smaller relative cortical gray matter (GM) volumes and densities in several regions, such as the prefrontal cortex, anterior cingulate cortex, orbitofrontal cortex, cerebellum, and thalamus^7–10^. Although the mechanisms underlying this GM volume loss have not been fully understood, animal studies have suggested that nicotine exposure can have neurotoxic consequences such as significant cell loss and synaptic alterations^11,12^. Moreover, cigarette smoking involves exposure to a wide range of toxic compounds, such as carbon monoxide (CO), free radicals, and free oxygen species^13^. These substances can cause direct damage to neuronal and brain tissue, leading to cellular and oxidative harm. Additionally, smoking can negatively impact inflammatory, respiratory, and vascular systems, resulting in deficits in blood oxygen and nutrients to the brain^14,15^. Smoking has also been shown to alter resting-state functional connectivity (rsFC) in different large-scale networks, such as the default mode network (DMN) and the fronto-parietal network^16,17^; acute nicotine administration can boost cognitive performance by activating the salience network (SN) which, in turn, suppresses the task-negative, internally driven DMN, while momentarily enhancing the intrinsic connectivity of the executive control network (ECN)^18^. However, protracted exposure to nicotine has been widely associated with disruptions in functional connectivity across circuits and large-scale networks, mostly involved in attention and cognitive control^19^. Since smoking behavior tends to be highly correlated with impulsivity, as numerous studies have previously reported^20–22^, it is often unclear how much tobacco consumption alone contributes to brain structural and functional differences. In this study, ninety-seven smokers and healthy non-smokers with comparable levels of impulsivity, as assessed through the Barratt Impulsiveness Scale (BIS-11)^23^, were selectively recruited; thereby, it was possible to have a clearer understanding of the effects of smoking on both structural and functional changes, without the confounding factor of impulsive behavior. Moreover, all participants were male and smoked for at least six months prior to the study, thus further increasing the homogeneity of the sample. By integrating functional and structural analyses, the present study aimed to provide further insights into the neural differences between smokers and non-smokers using a consistent demographic framework, with comparable values of age, education, impulsiveness^23^, and alcohol consumption^24^. Furthermore, it aimed to investigate both acute and chronic effects of tobacco use through a multifaceted and integrated assessment, employing comprehensive measures such as the CO level and the number of daily cigarettes. This way, it was possible to have a holistic understanding of the interplay between immediate and long-term effects of tobacco consumption on brain function and structure, setting our study apart in its contribution to unraveling the dynamics of smoking behavior. Based on previous findings of smoking-related research, we hypothesized that smokers would show altered rsFC and GM volume within different large-scale networks compared to non-smokers (e.g., dorsal attention network, central executive network), especially in relation to attentional processes and response inhibition, and that these differences would be associated with the smoking variables taken into account. Specifically, we expected to find widespread connectivity changes—towards the increase or decrease of rsFC—associated with acute nicotine exposure, with fluctuating levels of alteration according to state-dependent variables such as the CO level. On the other hand, we expected to find decreases in GM volume, specifically located within the prefrontal, anterior cingulate, and orbitofrontal cortex, as a potential consequence of the neurotoxic effects of chronic smoking behavior.

## Methods

### Participants

Ninety-seven right-handed healthy male participants (52 smokers and 45 non-smokers) aged 19–50 were recruited via public advertising. All participants were screened for psychiatric disorders using the Structured Clinical Interview for DSM-5 (SCID-5-CV)^25^. Any contraindication for magnetic resonance imaging (MRI; e.g., metal implants) and current neurological or psychiatric disorders led to exclusion from study participation. Smokers and non-smokers did not differ with regard to age, education, impulsivity traits, and alcohol consumption (see Table 1). Further, no significant differences in total grey matter (*t*_*95*_ = −1.58, *p* = 0.059), total white matter (*t*_*95*_ = −0.49, *p* = 0.313), and total intracranial volume (*t*_*95*_ = −1.45, *p* = 0.075) were detected between smokers and non-smokers. The study protocol was approved by the internal review board of the medical faculty of the University Hospital RWTH Aachen and was in accordance with the most recent amendment of the Declaration of Helsinki. All participants gave written informed consent and were compensated for their participation. The research project is preregistered at the German Clinical Trials Register (https://drks.de/search/de/trial/DRKS00024471). Characteristics of the study sample are presented in Table 1.

**Table 1:** Demographic characteristics of smokers and healthy non-smokers.

	Smokers (*N* = 52)	Non-smokers (*N* = 45)
Age (years)	26.08 ± 6.91	27.16 ± 6.15
Education (years)	12.83 ± 1.84	12.43 ± 1.14
BIS-11	62.6 ± 9.34	62.24 ± 11.51
FTND	3.72 ± 2.18	NA
AUDIT	6.09 ± 4.08	6.82 ± 5.21
Number of years smoking*	8.93 ± 7.89	NA
Cigarettes per day	12.32 ± 4.48	NA
CO level (ppm)	3.8 ± 1.32	NA

All data are reported with mean ± standard deviation.

*BIS* Barratt Impulsiveness Scale, *FTND* Fagerström Test for Nicotine Dependence, *AUDIT* alcohol use disorders identification test, *CO* carbon monoxide, *ppm* parts per million.

*Information on the number of years smoking was available for forty-three out of fifty-two smokers.

### Design

Following the study instructions, participants completed the German versions of the Barrett Impulsiveness Scale (BIS-11)^23^, the Alcohol Use Disorders Identification Test (AUDIT)^24^ and the Fagerström Test for Nicotine Dependence (FTND)^26^. Subsequently, participants completed the Stop Signal Task (SST; data not included here) followed by a short break, during which smokers had a cigarette. Smokers were then asked to provide breath CO levels using a Smokerlyzer (Bedfont Scientific Ltd., Harrietsham, UK). The following MRI session included resting-state functional magnetic resonance imaging (fMRI), during which participants were instructed to look at a fixation cross and let their mind wander, and structural MRI.

### MRI data acquisition

MRI data were collected using a 3-Tesla Siemens PRISMA scanner (Siemens Medical Systems, Erlangen, Germany) located in the Department of Psychiatry, Psychotherapy and Psychosomatics, Medical Faculty, RWTH Aachen University Hospital. T1-weighted structural images were acquired with a 20-channel head coil by means of a three-dimensional magnetization-prepared rapid acquisition gradient echo image (MPRAGE) sequence (voxel size: 1 × 1 × 1 mm^3^; 256 × 256 matrix; field of view [FoV]: 256 × 256 mm^2^; 176 volumes; time repetition [TR] = 2300 ms; time echo [TE] = 2.98 ms; flip angle = 9°). Resting-state data were acquired using an echoplanar imaging (EPI) sequence with 34 slices. In-plane resolution of the slices was 64 × 64 pixels with a total field of view of 192 mm^2^ and a voxel-size of 3 × 3 × 3 mm^3^. Images were acquired with a TR of 2000 ms, a TE of 28 ms and a flip-angle of 77° in an interleaved sequence.

### MRI data preprocessing

Functional and anatomical data were preprocessed through CONN^27^ release 22.a^28^ toolbox implemented in SPM^29^ release 12.7771, using a flexible preprocessing pipeline^30^ including realignment with correction of susceptibility distortion interactions, outlier detection, direct segmentation and MNI-space normalization, and smoothing. Functional data were realigned using SPM realign & unwarp procedure^31^, where all scans were coregistered to a reference image (first scan of the first session) using a least squares approach and a 6 parameter (rigid body) transformation^32^, and resampled using b-spline interpolation to correct for motion and magnetic susceptibility interactions. Potential outlier scans were identified using ART^33^ as acquisitions with framewise displacement above 0.9 mm or global BOLD signal changes above 5 standard deviations^34,35^, and a reference BOLD image was computed for each subject by averaging all scans excluding outliers. Functional and anatomical data were normalized into standard MNI space, segmented into grey matter (GM), white matter (WM), and cerebrospinal fluid (CSF) tissue classes, and resampled to 3 mm isotropic voxels following a direct normalization procedure^35,36^ using SPM unified segmentation and normalization algorithm^37,38^ with the default IXI-549 tissue probability map template. Last, functional data were smoothed using spatial convolution with a Gaussian kernel of 8 mm full-width-half-maximum (FWHM). In addition, functional data were denoised using a standard denoising pipeline^39^ including the regression of potential confounding effects characterized by white matter timeseries (5 CompCor noise components), CSF timeseries (5 CompCor noise components), motion parameters and their first order derivatives (12 factors)^40^, outlier scans (below 102 factors)^33^, session effects and their first order derivatives (2 factors), and linear trends (2 factors) within each functional run, followed by bandpass frequency filtering of the BOLD timeseries^41^ between 0.008 Hz and 0.09 Hz. CompCor^42,43^ noise components within white matter and CSF were estimated by computing the average BOLD signal as well as the largest principal components orthogonal to the BOLD average, motion parameters, and outlier scans within each subject's eroded segmentation masks. From the number of noise terms included in this denoising strategy, the effective degrees of freedom of the BOLD signal after denoising were estimated to range from 36.7 to 70.2 (average 69.1) across all subjects^34^.

### First-level analysis

Group-level independent component analyses (group-ICA^44^) were performed to estimate 20 temporally coherent networks from the fMRI data combined across all subjects. The suggested number of components was determined by the developers of the CONN toolbox to ensure sufficient characterization and distinct separation of the represented components. This is achieved by matching the component to a network template through an automated spatial correlation process^27^ and is in line with previous studies using low model order analysis^45–47^. The BOLD signal from every timepoint and voxel in the brain was concatenated across subjects and conditions along the temporal dimension. A singular value decomposition of the z-score normalized BOLD signal (subject-level SVD) with 64 components separately for each subject was used as a subject-specific dimensionality reduction step. The dimensionality of the concatenated data was further reduced using a singular value decomposition (group-level SVD) with 20 components, and a fast-ICA fixed-point algorithm^48^ with hyperbolic tangent (G1) contrast function was used to identify spatially independent group-level networks from the resulting components. Last, GICA3 back-projection^49^ was used to compute ICA maps associated with these same networks separately for each individual subject.

### Group-level analyses

Group-level analyses were performed using a General Linear Model (GLM^50^). For each individual voxel a separate GLM was estimated, with first-level connectivity measures at this voxel as dependent variables (one independent sample per subject), group as the independent variable, and age and total intracranial volume (TIV) as covariates. Voxel-level hypotheses were evaluated using multivariate parametric statistics with random-effects across subjects and sample covariance estimation across multiple measurements. Inferences were performed at the level of individual clusters (groups of contiguous voxels). Cluster-level inferences were based on parametric statistics from Gaussian Random Field Theory^51,52^. Results were thresholded using a combination of a cluster-forming *p* < 0.001 voxel-level threshold, and a family-wise corrected p-FWE < 0.05 cluster-size threshold^53^.

### Structural analysis

All T1-weighted images were first visually scanned for imaging artifacts or any other abnormalities. One subject had to be excluded from further analyses due to a structural abnormality. T1-weighted MRI scans were then analyzed with the Computational Anatomy Toolbox (CAT12; www.neuro.uni-jena.de/cat/)^54^ implemented in SPM^29^. Image preprocessing followed the default settings of CAT12; all images were segmented into grey matter (GM), white matter (WM), and cerebrospinal fluid (CSF) components and spatially normalized within the Montreal Neurological Institute (MNI) template. Afterward, TIV was estimated, and the homogeneity of the sample was checked for using the unsmoothed segmentations. Quartic mean z-scores were corrected for TIV by global scaling. Finally, spatial smoothing with an 8 mm full-width-half-maximum (FWHM) Gaussian kernel was applied to minimize the potential misalignment and enhance the signal-to-noise ratio. To examine the difference in GM volume between smokers and non-smokers, an independent sample* t*-test was conducted, including age as a covariate of no interest, and correcting for TIV to account for different brain sizes. Multiple regression analyses were performed within the smokers’ group to detect relations between GM volume and magnitude of tobacco consumption, here operationalized as the number of daily cigarettes and the CO level. For all analyses, absolute masking with a threshold of 0.1 was applied to ensure that only the intended tissue type was analyzed. A statistical threshold of *p* < 0.05 corrected for multiple comparisons using the Family-wise Error (FWE) rate on cluster-level was also applied.

## Results

### Functional connectivity (ICA)

**Figure 1:**
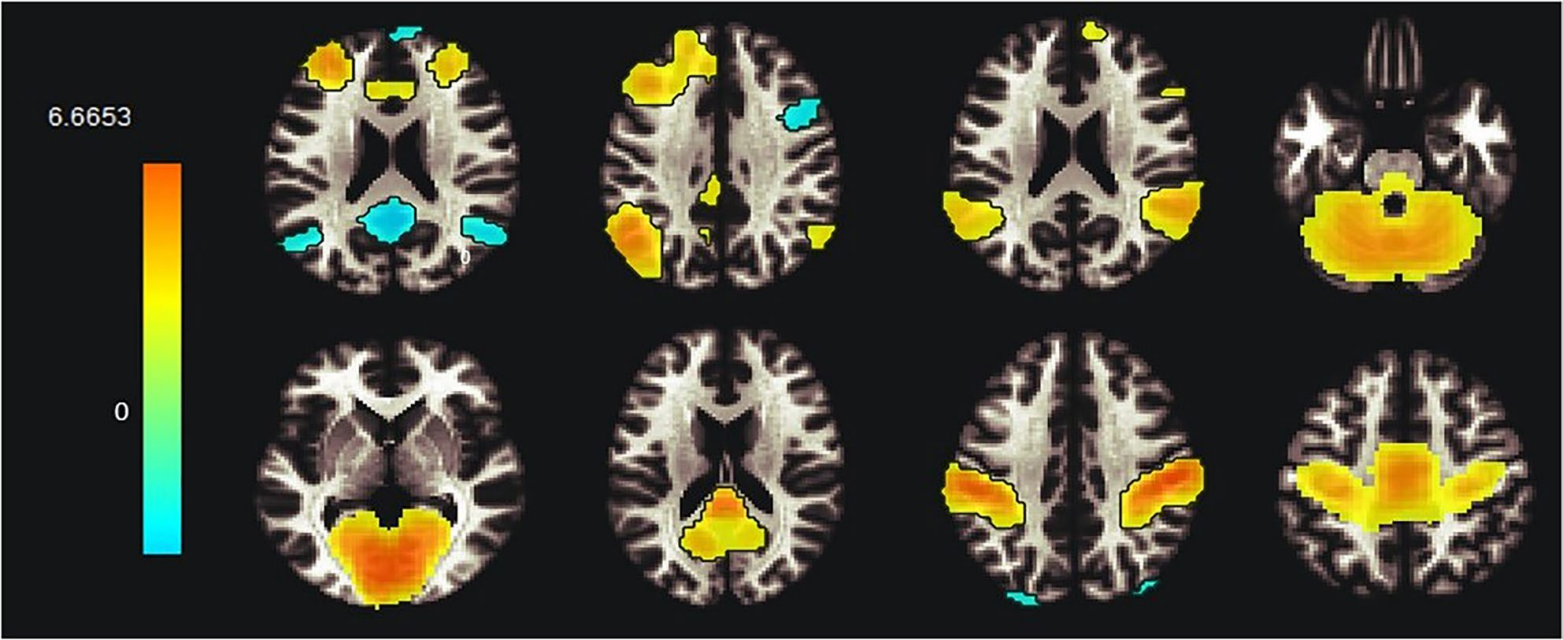
Identified resting-state networks within the group-ICA analysis (in order: salience, fronto-parietal, language, cerebellar, visual, default mode, dorsal attention, sensorimotor). The color bar indicates *t*-values with increased connectivity (red/yellow) and decreased connectivity (blue/green).

**Figure 2:**
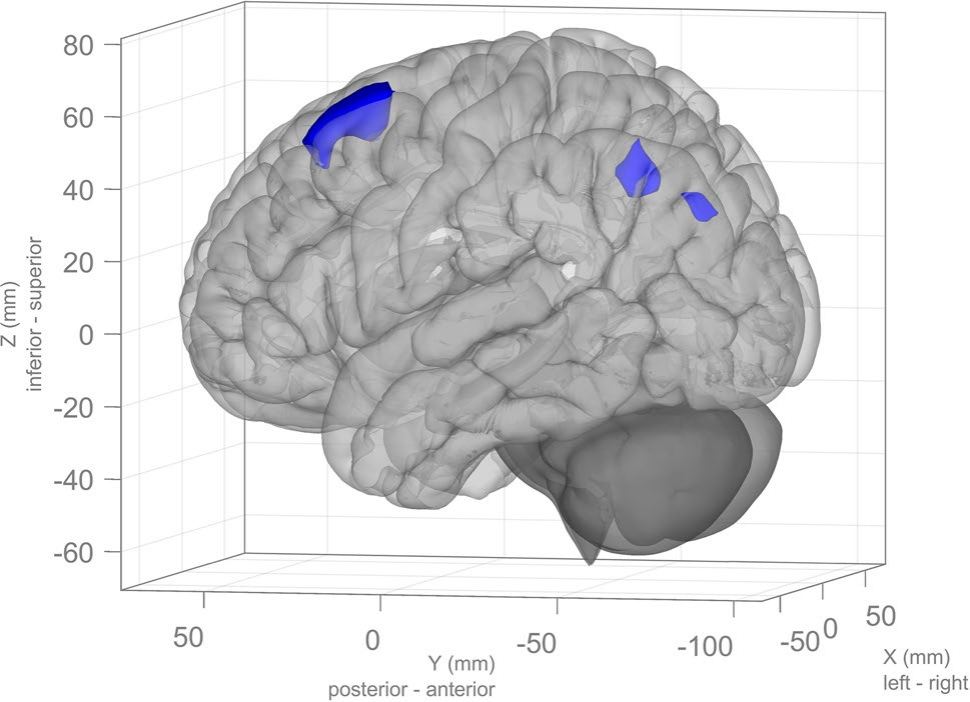
The left superior and middle frontal gyrus (*x* = -26, *y* =  + 12, *z* =  + 62, size = 522) and left superior division of the lateral occipital cortex (*x* = −36, *y* = −66, *z* =  + 42, size = 257) show decreased functional connectivity within the dorsal attention network in smokers compared to healthy non-smokers.

A spatial match-to-template revealed correlation (Pearson’s *r*) values for each of the 20 estimated independent components with resting-state networks within the CONN toolbox (i.e., salience, fronto-parietal, language, cerebellar, visual, default mode, dorsal attention, sensorimotor). Based on the highest correlation between each group spatial map and templates of resting-state networks, eight components—one for each resting-state network—were identified and extracted using a group-ICA (salience network: *r* = 0.37; fronto-parietal network: *r* = 0.26; language network: *r* = 0.34; cerebellar network: *r* = 0.31; visual network: *r* = 0.51; default mode network: *r* = 0.36; dorsal attention network: *r* = 0.54; sensorimotor network: *r* = 0.46; see Fig. 1). They were then compared between the two groups (smokers *vs* non-smokers) using an independent sample *t*-test. Smokers showed reduced connectivity within the dorsal attention network (DAN) in the left superior and middle frontal gyrus (MNI coordinates: *x* = −26, *y* =  + 12, *z* =  + 62, size = 522; T(94) = -5.26, *p*-FWE_cluster-level_ < 0.001) and left superior division of the lateral occipital cortex (MNI coordinates: *x* = −36, *y* = -66, *z* =  + 42, size = 257; T(94) = -4.99, *p*-FWE_cluster-level_ < 0.001), as shown in Fig. 2. Within smokers, higher levels of CO were associated with increased connectivity within the sensorimotor network (SMN) in the left central opercular cortex. Smokers with higher consumption of daily cigarettes, on the other hand, showed increased connectivity within the cerebellar network (CN) in the left and right cerebellum and within the default mode network (DMN) in the left frontal pole; they also showed decreased connectivity within the visual network (VN) in the left paracingulate gyrus and within the salience network (SN) in the anterior cingulate gyrus (see Table 2). Moreover, a positive significant correlation between CO levels and daily cigarette consumption was found (*ρ* = 0.417, *p* = 0.004).

**Table 2:** Regions with altered functional connectivity in smokers associated to CO level and daily cigarette consumption.

Covariate	Resting-state network	Region	Size	MNI coordinates (x, y, z)	Size p-FWE
CO	SMN ↑	L central opercular cortex	413	−60, + 00, + 02	< .001
Daily cigarettes	CN ↑	Bilateral cerebellum	614	−02, −42, −66	< .001
	SN ↓	Anterior cingulate gyrus	328	+ 06, + 08, + 38	< .01
	VN ↓	L paracingulate gyrus	208	−12, + 48, −10	< .05
	DMN ↑	L frontal pole	189	−20, + 66, + 12	< .05

↑ indicates increased connectivity, while ↓ indicates decreased connectivity.

*L* left, *SMN *sensorimotor network, *CN* cerebellar network, *SN* salience network, *VN* visual network, *DMN* default mode network, *MNI* montreal neurological institute, *FEW* family-wise error.

### Structural analysis

#### Voxel-based morphometry (VBM)

**Figure 3:**
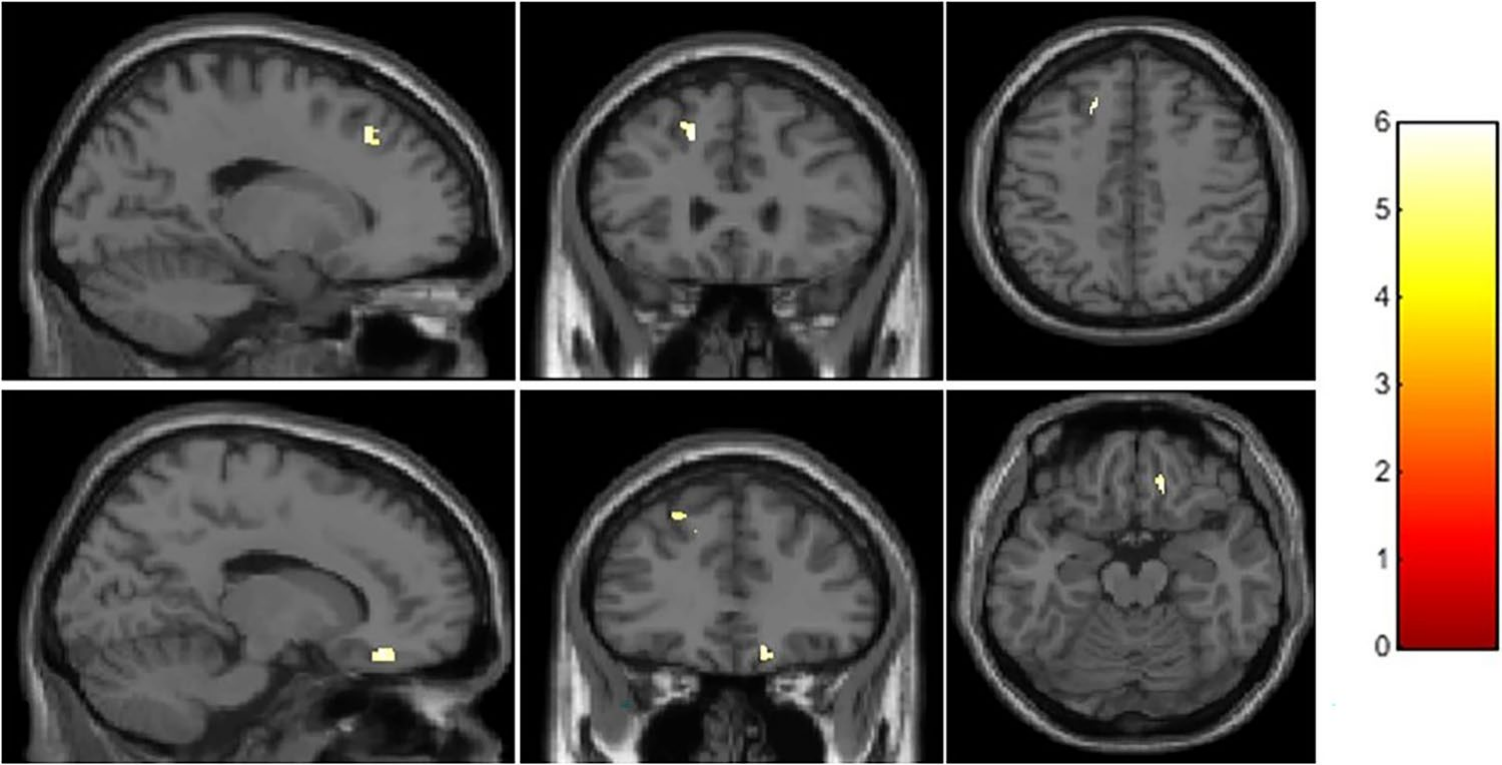
Statistical parametric maps overlaying averaged T1-weighted images show significantly smaller grey matter volume in the left superior and middle frontal gyrus (*x* = −15, *y* = 29, *z* = 42, cluster extent = 110) and right orbitofrontal cortex (*x* = 15, *y* = 35, *z* = −18, cluster extent = 54) in smokers compared with healthy non-smokers.

In comparison to healthy non-smokers, smokers showed significantly decreased GM volume in the left superior and middle frontal gyrus (MNI coordinates: *x* = −15, *y* = 29, *z* = 42, cluster extent = 110; T_peak-level_ = 5.79, *p*-FWE_peak-level_ < 0.01; *p*-FWE_cluster-level_ < 0.01) and in the right orbitofrontal cortex (MNI coordinates: *x* = 15, *y* = 35, *z* = −18, cluster extent = 54; T_peak-level_ = 5.95, *p*-FWE_peak-level_ = 0.001; *p*-FWE_cluster-level_ < 0.01), as shown in Fig. 3. Moreover, increasing age in smokers was associated with GM volume loss in widespread and extended brain regions. On the other hand, non-smokers showed a decreased GM volume with increasing age in a few areas (see Table 3). No regions were found where the GM volume was significantly correlated with the number of daily cigarettes, nor with the CO level.

**Table 3:** Regions with decreased grey matter volume associated with increasing age in smokers and healthy non-smokers.

Group	Region	Size	MNI coordinates (x, y, z)	Size p-FWE	*Z*-score
Smokers	R superior temporal sulcus	744	46.5, − 33, 9	< .001	6.23473
	L superior frontal gyrus	170	− 22.5, 9, 49.5	< .001	5.52216
	L *pars orbitalis*	205	− 48, 52.5, − 9	< .001	5.46216
	R rostral middle frontal gyrus	48	42, 36, − 13.5	< .01	5.39401
	R parahippocampal gyrus	58	18, − 36, − 9	< .01	5.20859
	R frontal pole	22	10.5, 66, − 7.5	< .05	5.07721
	R middle temporal gyrus	21	64.5, − 27, − 13.5	< .05	5.07263
	L superior temporal gyrus	12	− 48, − 13.5, − 4.5	< .05	5.06362
	L frontal *operculum*	28	− 43.5, 18, − 1.5	< .01	5.02166
	L *pars triangularis*	81	− 43.5, 37.5, 16.5	< .01	5.02065
	L middle frontal gyrus	18	− 51, 16.5, 31.5	< .05	4.99003
	R *pars orbitalis*	59	34.5, 61.5, 10.5	< .01	4.96308
	R superior frontal gyrus	21	10.5, 54, 30	< .05	4.91427
	L rostral middle frontal gyrus	10	− 13.5, 61.5, 0	< .05	4.91418
	L superior temporal gyrus	10	− 48, 4.5, − 3	< .05	4.79388
Non− smokers	Bilateral superior frontal gyri	103	0, 37.5, 36	< .001	5.317041
	L superior temporal gyrus	13	− 39, 10.5, − 34.5	< .05	4.970372
	L insula	25	− 30, 7.5, − 9	< .05	4.913628
	L posterior cingulate gyrus	18	− 4.5, − 33, 48	< .05	4.881205
	R putamen	13	31.5, − 1.5, 1.5	< .05	4.764697

*L* left, *R* right, *MNI* montreal neurological institute, *FEW* family-wise error.

## Discussion

In this study, we investigated the effect of cigarette smoking on the functional connectivity of eight standard resting-state networks, as well as structural GM differences between smokers and healthy non-smokers. Our results showed that smokers had decreased functional connectivity within the dorsal attention network (DAN) in the left superior and middle frontal gyrus and lateral occipital cortex compared to non-smokers, and these regions also partly overlapped with reduced GM volume in cigarette users. We also investigated the association between increasing age and GM volume loss in smokers and non-smokers separately and the effects of CO level and daily cigarette consumption on rsFC within the smokers’ group.

When inspecting the resting-state networks identified via the group-ICA we found reduced functional connectivity in smokers solely within the DAN, specifically in the left superior and middle frontal gyrus and left superior division of the lateral occipital cortex, in comparison to healthy non-smokers. The DAN is a large-scale brain network that, among other functions, encodes and maintains preparatory signals, participates in the control and initiation of voluntary movements, and modulates top-down activity during the spatial orienting of attention^55^. Previous studies have found that cigarette smoking has a negative impact on prefrontal attentional network functioning, causing less extended network activity in different areas, such as the supplementary motor area, anterior cingulate cortex, dorsolateral prefrontal cortex (including the middle frontal gyrus), and parietal cortex^56^. In fact, even though acute administration of nicotine is known to have positive effects on cognitive functioning, such as fine motor skills, working memory, and selective attention^5,57^, chronic cigarette smoking, on the other hand, is associated with lower cortical perfusion in multiple brain regions^58^. Since every study has a different procedure—smokers may or may not be allowed to smoke a cigarette before entering the scanner—it can be difficult to differentiate between acute and chronic effects of nicotine. Moreover, it has been consistently reported that chronic smokers might be subject to the neurotoxic effects of nicotine and other chemical compounds (e.g., carbon monoxide, free radicals, nitrogen oxides)^59^, which are closely related to GM volume loss^13^. Several studies have previously reported reduced GM volume in specific structures of the left hemisphere, including the insula, dorsal anterior cingulate cortex, and superior frontal gyrus^7,10,60,61^. Our results show overlapping functional and structural brain differences in smokers, with reduced GM volume and functional connectivity in the left superior and middle frontal gyrus, a region involved in, among other functions, response inhibition and attentional reorienting^62,63^. This overlap indicates that cigarette smoking may modify functional connectivity in the prefrontal areas, and goes along with parallel GM volume loss in that same region; in fact, it has been previously described how distributed network maps can mirror the cortical atrophy patterns seen in different forms of neurodegeneration^64^. As earlier reported by Pariyadath et al.^65^, rsFC within the middle superior frontal gyrus has been identified as a discriminatory feature in a machine-learning classifier to distinguish between smokers and non-smokers, which could potentially be predictive of nicotine dependence; changes within this network might therefore serve as a circuit-based biomarker for future structural deficits^6^. Furthermore, we found that age seems to affect smokers and healthy non-smokers differently, although it must be mentioned that the mean age of both groups is moderately young, as reported in Table 1. Specifically, age among smokers has a large, widespread effect on GM volume loss, while among non-smokers this effect is limited to a few clusters. According to Durazzo et al.^66,67^, smokers showed greater volume loss of anterior frontal regions such as the rostral middle frontal gyrus, medial orbitofrontal cortex, *pars triangularis*, *pars orbitalis*, lateral orbitofrontal cortex, frontal pole, and posterior regions associated with neuroanatomic abnormalities in Alzheimer's disease, including the hippocampus (included all subregions), inferior parietal lobule, and middle temporal gyrus for each year of advancing age compared to healthy non-smokers. Our results seem to be in line with these previous findings.

Within the smokers’ group, we discovered that breath CO concentration and daily cigarette consumption seem to measure different aspects of smoking behavior since they tend to have differential influences on rsFC. In our study, the CO level was associated with increased resting-state connectivity within the SMN, while daily cigarette consumption was positively associated with increased connectivity within the CN and the DMN, and negatively associated with the SN and the VN. Nevertheless, neither CO level nor daily cigarette consumption showed a significant effect on GM volume loss. While CO level has been reported as an immediate and effective measure of smoking status^68^, mostly related to state-dependent variables (e.g., puff volume^69^, inhalation pattern^70^, filter vent blocking^71^), the number of daily cigarettes does not capture the effects of smoking topography^72^ and vent blocking^71^. Moreover, CO level can vary according to pre- and post-cigarette measurements; this difference is known as CO boost, which has been shown to be positively associated with total and average puff volume, depth of inhalation, and puff duration^69,73^. On the other hand, daily cigarette consumption, being a self-report measure, solely relies on the participants' recalling; for this reason, it may be an inaccurate measure of smoking intensity^74^, but it is not susceptible to rapid changes in smoking behavior. Since they seem to affect different brain networks, both indicators should therefore be used to better assess tobacco consumption.

The following limitations of the study should be reported. First, since the sample is constituted of only males to increase the homogeneity of the sample itself, the results presented here are not generalizable to the female population. Secondly, measures of CO level were taken at different times during the day for each participant, and this may have contributed to higher variability in the smokers' breath CO concentration. Finally, it was not possible to make direct comparisons between smokers' and non-smokers' age groups but only to see the effect of age on both populations separately. Future studies should focus more on this aspect, using functional and structural analyses to investigate the effects of smoking—according to different variables, including smoking topography, filter vent blocking, and age—on rsFC and GM volume.

## Conclusion

The present study shows that cigarette smokers, compared to non-smokers, exhibit decreased functional connectivity in the left superior and middle frontal gyrus and left lateral occipital cortex, regions that are heavily involved in attentional processes and response inhibition. Smokers also presented reduced GM volume in the left superior and middle frontal gyrus, showing overlap with rsFC findings, and in the right orbitofrontal cortex. Even though the causality of the relationship cannot be directly inferred, our results indicate that smoking might be negatively related to rsFC and GM volume, notably within prefrontal areas, thus offering further insight into the neural substrates of smoking behavior.

## Data Availability

The data analyzed during the current study will be made available from the corresponding author upon reasonable request.
